# Prognostic power of a lipid metabolism gene panel for diffuse gliomas

**DOI:** 10.1111/jcmm.14647

**Published:** 2019-09-01

**Authors:** Fan Wu, Zheng Zhao, Rui‐Chao Chai, Yu‐Qing Liu, Guan‐Zhang Li, Hao‐Yu Jiang, Tao Jiang

**Affiliations:** ^1^ Department of Molecular Neuropathology Beijing Neurosurgical Institute Capital Medical University Beijing China; ^2^ Department of Neurosurgery Beijing Tiantan Hospital Capital Medical University Beijing China; ^3^ Chinese Glioma Genome Atlas Network (CGGA) and Asian Glioma Genome Atlas Network (AGGA) Beijing China

**Keywords:** diffuse glioma, lipid metabolism, prognosis, progression, signature

## Abstract

Lipid metabolism reprogramming plays important role in cell growth, proliferation, angiogenesis and invasion in cancers. However, the diverse lipid metabolism programmes and prognostic value during glioma progression remain unclear. Here, the lipid metabolism‐related genes were profiled using RNA sequencing data from The Cancer Genome Atlas (TCGA) and Chinese Glioma Genome Atlas (CGGA) database. Gene ontology (GO) and gene set enrichment analysis (GSEA) found that glioblastoma (GBM) mainly exhibited enrichment of glycosphingolipid metabolic progress, whereas lower grade gliomas (LGGs) showed enrichment of phosphatidylinositol metabolic progress. According to the differential genes of lipid metabolism between LGG and GBM, we developed a nine‐gene set using Cox proportional hazards model with elastic net penalty, and the CGGA cohort was used for validation data set. Survival analysis revealed that the obtained gene set could differentiate the outcome of low‐ and high‐risk patients in both cohorts. Meanwhile, multivariate Cox regression analysis indicated that this signature was a significantly independent prognostic factor in diffuse gliomas. Gene ontology and GSEA showed that high‐risk cases were associated with phenotypes of cell division and immune response. Collectively, our findings provided a new sight on lipid metabolism in diffuse gliomas.

## 1 INTRODUCTION

Recently, metabolic reprogramming has been recognized as a new hallmark of cancer cells.^1^ Increased glycolysis under normoxic condition (Warburg effect) and glutamine metabolism are the main features of malignant tumours.^2^, ^3^ Now, the deregulation of lipid metabolism has also been considered as one of the most important metabolic hallmarks of cancer cells. Highly proliferative cancer cells can acquire lipids by enhancing lipid uptake, lipolysis and de novo fatty acid synthesis.^4^

Glioma is one of the most treatment‐refractory cancers and highly resistant to chemo and radiotherapy.^5^ Most diffuse LGGs and nearly all glioblastomas will eventually recur and often transform into a higher grade. It has reported that unsaturated fatty acid, cholesterol esters and phosphatidylcholine are only present in high‐grade gliomas through magnetic resonance spectroscopy (NMR) analysis.^6^, ^7^ At present, more and more studies focus on revealing the biological phenotype and molecular mechanism that altered lipid component leads to in glioma. Offer et al found that extracellular lipid loading augments hypoxic paracrine signalling and promotes glioma angiogenesis and macrophage infiltration.^8^ *GPIHBP1*, a GDP‐anchored protein of capillary endothelial cells, facilitated triglyceride‐rich lipoproteins (TRLs) processing and provided a source of lipid nutrients for glioma cells.^9^ Marifia and colleagues revealed that sphingosine‐1‐phosphate (*S1P*) fuelled proliferative and stemness qualities of glioblastoma stem cells.^10^ However, the distinct lipid metabolism programmes and prognostic value in glioma progression need further study.

In this study, we profiled the lipid metabolism status in 859 diffuse glioma samples with gene expression data from TCGA and CGGA database. Distinct enrichments of lipid metabolism phenotype were observed between LGGs and GBM. Then, we constructed a lipid metabolism‐related gene set for evaluating the risk of poor outcome, which was also validated in CGGA cohort. The gene set was closely associated with the pathological factors and could be identified as an independent prognostic feature. Taken together, our results indicated a strong connection between patients' survival and lipid metabolism in diffuse glioma.

## 2 METHODS

### 2.1 Patients and datasets

We collected 550 and 309 diffuse gliomas with RNA‐seq data and clinical information from TCGA and CGGA database, respectively.^11^, ^12^ TCGA cohort was used as training set and CGGA cohort as validation set. All tissues and clinicopathologic information were obtained with written informed consents. This study was approved by ethics committee of Tiantan Hospital. The patient characteristics of these two cohorts were summarized in Table S1.

### 2.2 Gene set selection

Four lipid metabolism‐related gene sets (Reactome metabolism of lipids and lipoproteins, Reactome phospholipid metabolism, Hallmark fatty acid metabolism and KEGG glycerophospholipid metabolism) were collected from the Molecular Signature Database v5.1 (MSigDB).^13^ After removing the overlapped genes, 614 lipid metabolism‐related genes were obtained. The differential lipid metabolism genes between LGG and GBM were selected. By using the R package ‘survival’, univariate Cox analysis performed to prefilter the genes based on the *P* values. Then, the Cox proportional hazards model with elastic net penalty was applied for selecting signature gene, which was performed with the R package ‘glmnet’.^14^, ^15^ A linear combination of signature genes expression level weighted by regression coefficients (Coeffs) was developed to calculate the risk score of each patient in training set. Then, the regression Coeffs from training set was used to compute the risk scores for cases of validation set.

### 2.3 Bioinformatic analysis

Gene ontology (GO) analysis was performed for function annotation of differential genes.^16^ Gene set enrichment analysis (GSEA) was applied for identifying statistically different gene sets between two groups with GSEA v3 software.^13^ Principal components analysis (PCA) was carried out using the R package ‘princomp’ to analyse the expression pattern of grouped patients.^17^, ^18^ Utilizing the gene expression data, stromal and immune score of each sample was calculated with R package ‘ESTIMATE’ which reflected the gene signature enrichment of stromal and immune cells.^19^

### 2.4 Statistical analysis

Patients in both training and validation cohorts were assigned into high‐ or low‐risk group based on the median value of risk score. Kaplan‐Meier curves and 2‐sided log‐rank test were applied to assess the survival difference between high‐ and low‐risk groups. Chi‐square test was conducted to detect the pathologic differences between high‐ and low‐risk patients. Univariate and multivariate Cox regression analyses were performed to assess the independent prognostic factors by using SPSS software. ROC curve analysis was used to predict overall survival (OS) with R package ‘pROC’. *P* value <.05 was considered significant statistically.

## 3 RESULTS

### 3.1 LGG and GBM show distinct lipid metabolism phenotypes

**Figure 1:**
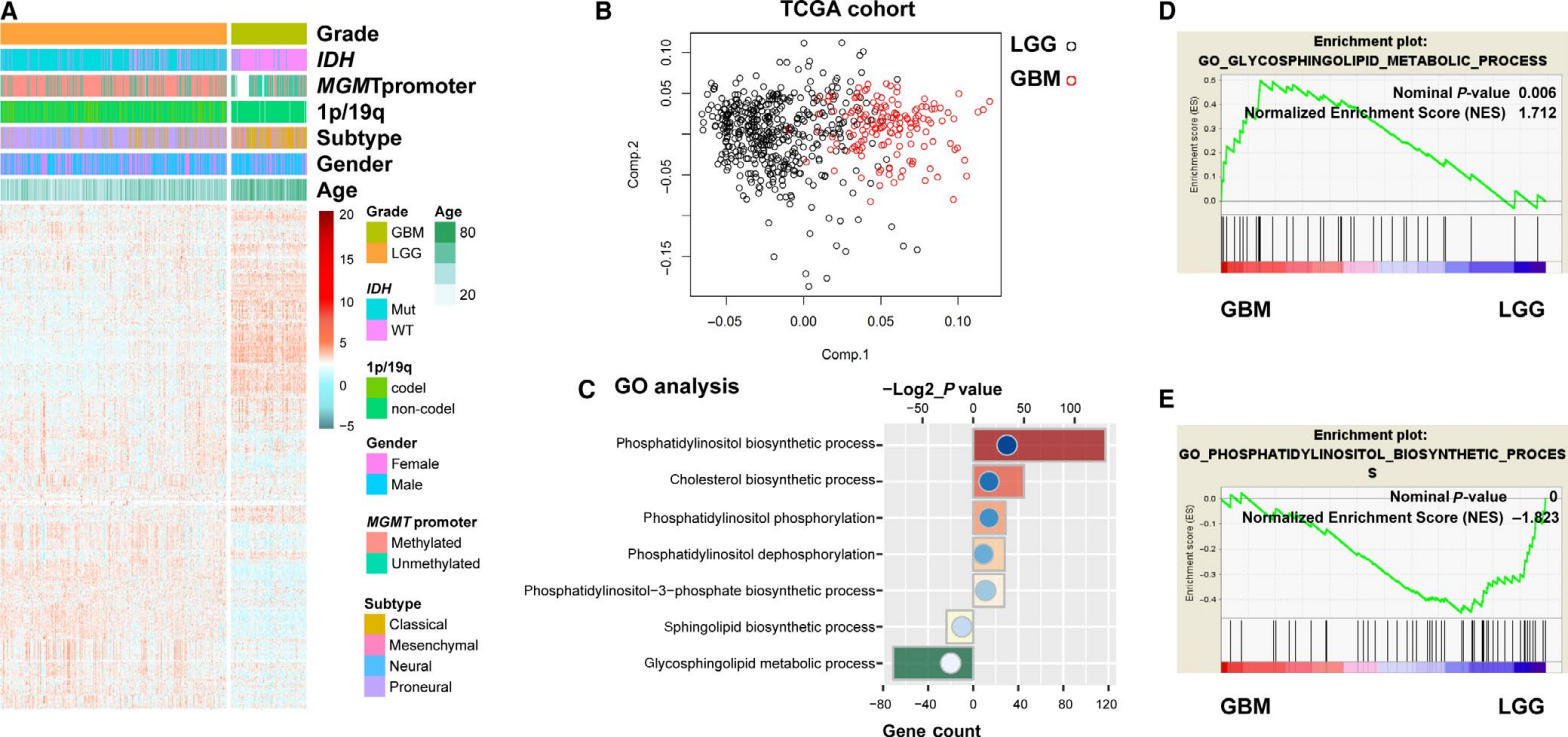
Distinct lipid metabolism status between LGG and GBM. A, Heat map of lipid metabolism‐related genes between LGG and GBM of TCGA cohort. B, Principal components analysis of lipid metabolism‐related genes between LGG and GBM. C, GO analysis of differential genes between LGG and GBM. D and E, Gene set enrichment analysis of lipid metabolism status between LGG and GBM. NES, normalized enrichment score

To detect the lipid metabolism differences during the progression of diffuse gliomas, we collected 550 patients with RNA sequencing data and clinical information from TCGA database and four lipid metabolism‐related gene sets, which were integrated into one set containing 614 genes. Gene clustering using the R package ‘pheatmap’ found that the profile of lipid metabolism‐related genes between LGG and GBM showed obvious differences (Figure 1A). Principal components analysis based on these selected genes showed that GBM and LGG were distributed in different regions, suggesting distinct lipid metabolism phenotypes between them (Figure 1B). To further explore the lipid metabolism phenotypes, we performed GO analysis and found that GBM mainly exhibited an enrichment of glycosphingolipid metabolic progress, whereas LGG displayed enrichment of phosphatidylinositol metabolic progress (Figure 1C). Gene set enrichment analysis analysis also confirmed this finding (Figure 1D,E). In addition, we also analysed the CGGA cohort of 309 glioma samples using the above methods, and the same results were observed between LGG and GBM (Figure S1). Heat maps showed the differential genes between LGG and GBM, involving in glycosphingolipid and phosphatidylinositol metabolic progress (Figure S2). These results indicated LGG and GBM displayed distinct lipid metabolic phenotypes.

### 3.2 Identification of a lipid metabolism‐related gene set for prognostic prediction

**Figure 2:**
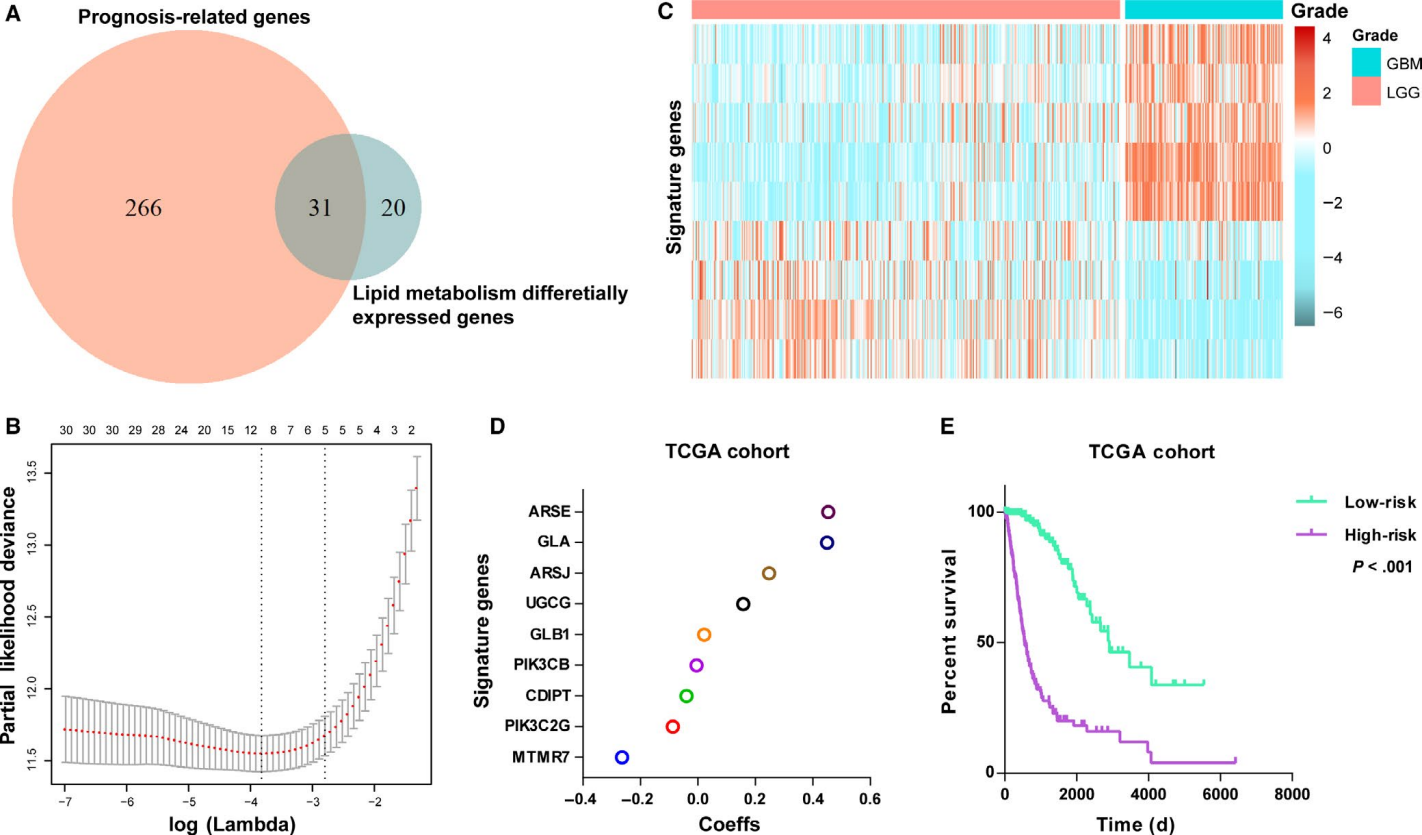
Identification of a prognostic signature by Cox proportional hazards model in TCGA cohort. A, Venn diagram shows prognosis‐related lipid metabolism genes which are also differentially expressed between GBM and LGG. B, Cross‐validation for tuning parameter selection in the proportional hazards model. C, Heat map shows the signature genes. D, Coefficient (Coeff) values of the nine selected genes. E, Survival analysis of OS in high‐ and low‐risk groups of patients

Considering the distinct profile of lipid metabolism between LGG and GBM, we proposed to build a lipid metabolism‐related gene set for predicting prognosis. By performing univariate Cox regression analysis, 297 prognosis‐related genes remained (*P* < .05). Thirty‐one out of prognosis‐related genes were involved in glycosphingolipid and phosphatidylinositol metabolic progress (Figure 2A). Then, we performed the Cox proportional hazards model with elastic net regression for gene selection (Figure 2B). Consequently, a nine‐gene signature was obtained as a classifier (Figure 2C,D), and risk score of each patient was computed with expression value and the coeffs of multivariable Cox regression.

Then, based on the median risk score, patients were assigned into high‐ or low‐risk group. Kaplan‐Meier analysis found the high‐risk cases had a significantly shorter OS than low‐risk ones (*P* < .001, Figure 2E). To validate this gene set, we also calculated patients' risk scores of CGGA cohort with same regression Coeffs. Heat map showed the expression of signature genes in CGGA cohort (Figure S3A). As expected, we acquired consensus result (Figure S3B).

### 3.3 The nine‐gene set shows strong prognostic power

We next performed univariate and multivariate Cox regression analyses to determine the prognostic value of the acquired gene set. The results showed that the lipid metabolism‐related gene set was independently correlative with OS (*P* = .017) (Table 1). Consistently, this gene set could also be served as an independent prognostic factor in CGGA validation set (*P* = .003) (Table 1). By computing the AUC of risk score, age and grade, we next assessed the predictive accuracy with ROC curve and found that AUC of risk score (0.86) was much higher than that of age (0.801) or grade (0.83) (Figure S4A). Similar results were also observed in CGGA validation set (Figure S4B). These results indicated that the acquired lipid metabolic gene set had strong power for prognosis prediction.

**Table 1:** Univariate and multivariate Cox regression analysis of clinical pathologic features for OS in TCGA and CGGA cohorts

Characteristics	TCGA cohort						CGGA cohort					
	Univariate analysis			Multivariate analysis			Univariate analysis			Multivariate analysis		
	HR	95% CI	*P*‐value	HR	95% CI	*P*‐value	HR	95% CI	*P*‐value	HR	95% CI	*P*‐value
Age	1.076	1.063‐1.089	<**.001**	1.061	1.044‐1.078	<**.001**	1.038	1.022‐1.053	<**.001**	1.006	0.989‐1.077	.637
Gender	0.957	0.705‐1.299	.779				0.843	0.597‐1.189	.33			
Grade	5.285	4.047‐6.902	<**.001**	1.561	1.056‐2.309	**.026**	3.469	2.709‐4.443	<**.001**	1.961	1.355‐2.839	<**.001**
Subtype	2.398	2.038‐2.822	<**.001**	0.976	0.739‐1.289	.866	0.583	0.492‐0.691	<**.001**	0.872	0.706‐1.077	.203
*IDH*	0.101	0.07‐0.144	<**.001**	0.841	0.363‐1.945	.685	0.229	0.159‐0.331	<**.001**	0.806	0.434‐1.494	.493
*MGMT* promoter	0.276	0.196‐0.39	<**.001**	0.885	0.594‐1.319	.55	0.529	0.374‐0.75	<**.001**	0.812	0.536‐1.23	.327
1p/19q	0.212	0.122‐0.367	<**.001**	0.433	0.227‐0.823	**.011**	0.165	0.067‐0.404	<**.001**	0.607	0.236‐1.563	.301
Risk score	2.433	2.14‐2.767	<**.001**	1.496	1.075‐2.083	**.017**	1.298	1.242‐1.355	<**.001**	1.132	1.044‐1.228	**.003**

Abbreviations: CI, confidence interval; HR, hazard ratio; *IDH*, isocitrate dehydrogenase; *MGMT*, methylguanine methyltransferase.

*P* value (<.05) marked in bold was considered significant statistically.

### 3.4 The acquired nine‐gene set is correlated with pathologic features in diffuse gliomas

**Figure 3:**
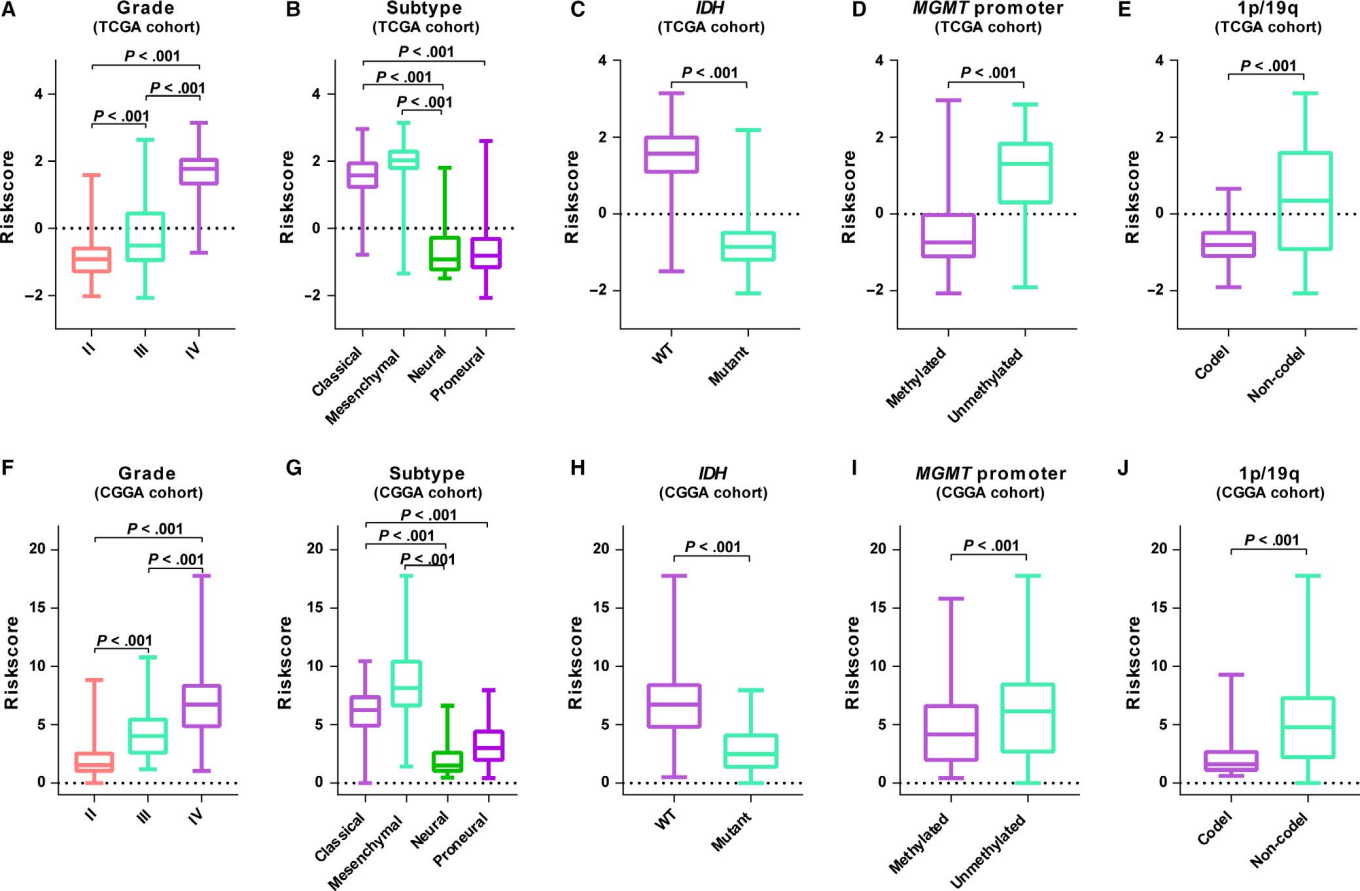
Association between the lipid metabolism‐related gene panel and pathologic features. A‐E, Distribution of the risk score in stratified patients by grade, subtype, *IDH*, *MGMT* promoter and 1p/19q status in TCGA cohort. F‐J, Distribution of the risk score in stratified patients by grade, subtype, *IDH*, *MGMT* promoter and 1p/19q status in CGGA cohort

We further detected whether the gene set was associated with pathologic features. As shown in Figure 3, higher level of risk scores preferred to distribute in higher grade, classical, mesenchymal, *IDH*‐wt, *MGMT* promoter unmethylated or 1p/19q non‐codeleted patients. We also assessed the distributive differences of these pathologic features between high‐ and low‐risk groups by performing chi‐square test. In both cohorts, most of pathologic features had significantly different distribution between risk groups except gender (Table S1). These results suggested a significant association between the lipid metabolism gene set and clinical molecular features.

### 3.5 Application of the nine‐gene panel in stratified patients

**Figure 4:**
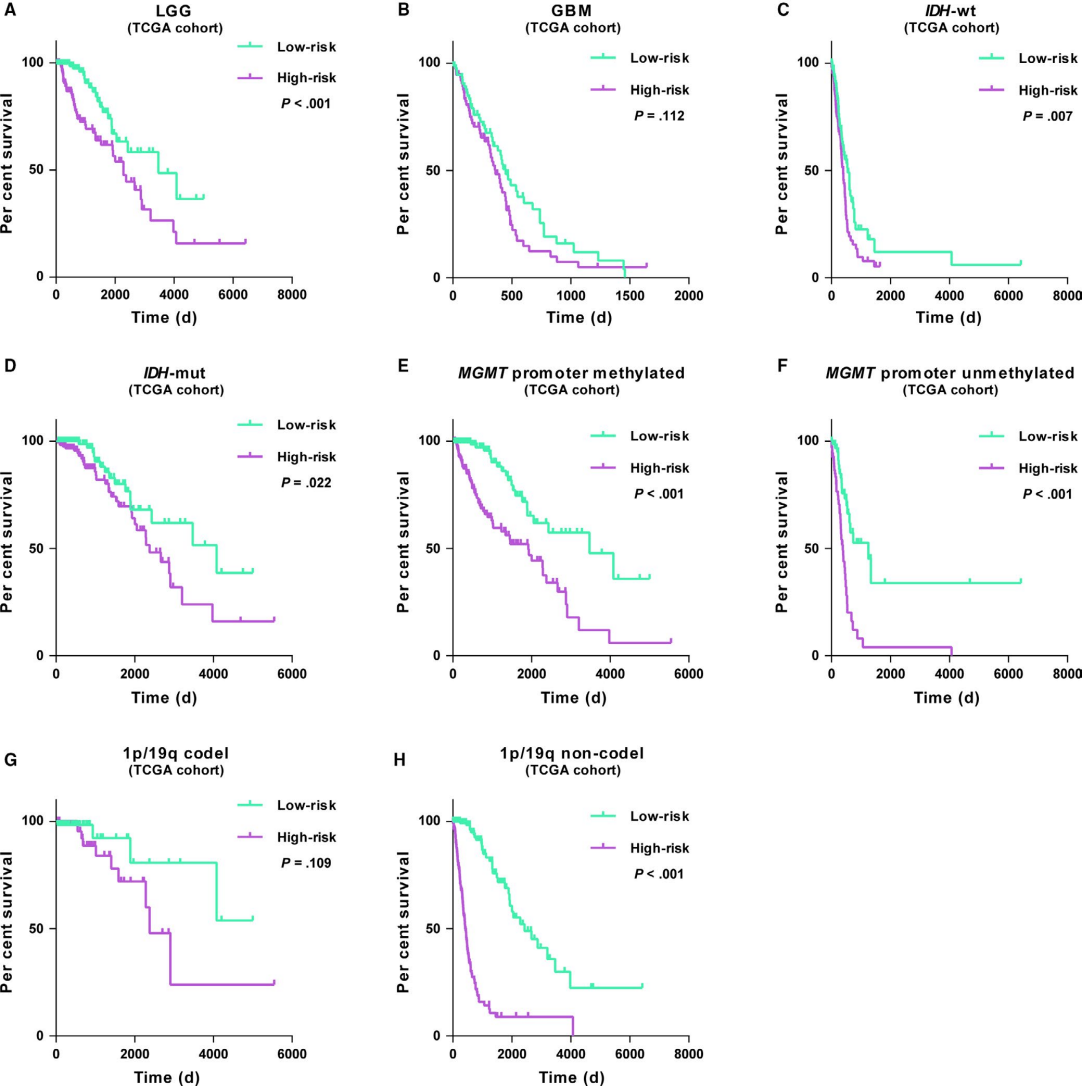
Prediction of outcome of the gene signature in stratified patients. A‐H, Survival analysis of the signature in patients stratified by grade, *IDH*, *MGMT* promoter and 1p/19q status

We further explored the prognostic significance of the gene panel in patients stratified by grade, *IDH*, *MGMT* promoter and 1p/19q status. In both cohorts, Kaplan‐Meier analysis showed that cases with high‐risk score had shorter overall survival than the low‐risk ones in most stratified patients (Figure 4, Figure S5). The similar trend occurred in GBM or 1p/19q codeleted cases despite of no statistical difference (Figure 4B,G). After that, patients were also stratified by WHO 2016 molecular subtype. Consensus results were obtained in cases of *IDH*‐mutant LGG, whereas in *IDH*‐wt LGG, *IDH*‐wt GBM and *IDH*‐mutant GBM found no significant differences (Figure S6). These data revealed that acquired signature could accurately predict the unfavourable outcome in most stratified patients.

### 3.6 High‐risk cases show enhanced cell division and immune response phenotypes

**Figure 5:**
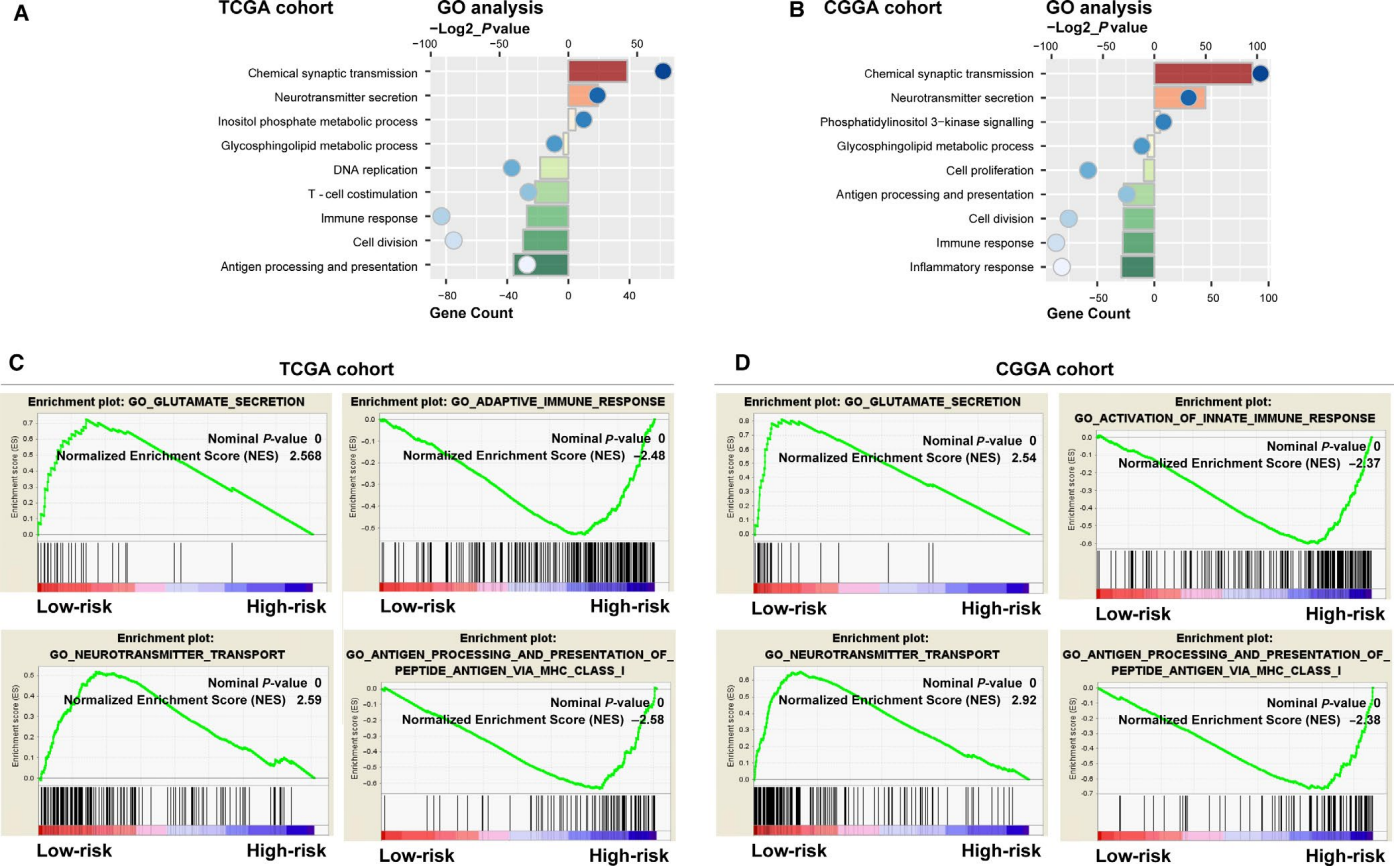
Functional enrichments between low‐ and high‐risk cases. A and B, GO analysis of differential genes between low‐ and high‐risk cases in two cohorts. C and D, GSEA analysis based on the median value of the risk scores in two cohorts

To detect the biological function differences, we further compared gene expression of patients between low‐ and high‐risk groups. PCA found that low‐ and high‐risk cases distributed in two regions clearly (Figure S7). Based on the differentially expressed genes (*P* < .05) which were identified by SAM, GO analysis found that cell division and immune response were significantly enriched in high‐risk patients, whereas low‐risk cases showed enrichments of chemical synaptic transmission and neurotransmitter secretion (Figure 5A,B). Moreover, GSEA also confirmed these findings (Figure 5C,D).

## 4 DISCUSSION

Compelling evidence has suggested that metabolism deregulation is one of the emerging hallmarks of cancer cells, due to its important role in cell growth, proliferation, angiogenesis and invasion. Warburg reported that cancer cells mainly obtain energy by shifting their metabolism towards glycolysis pathway rather than oxidative phosphorylation.^1^ In addition to the abnormal glucose metabolism, lipids, amino acids and nucleic acids metabolism are also altered in cancer cells.^20^ Recent studies have found that lipid metabolism reprogramming plays a crucial part in membrane synthesis, energetic production and signal transduction in the progression of cancer cells.^21^

Glioma, an intractable cancer, is one of the most lethal human brain malignancies with frequent recurrences 6 months after surgery. Although great efforts have made on the glucose metabolism alterations, increasing research has indicated that lipid metabolism is also aberrant in glioma. In addition to the increased lipid level,^6^, ^7^ the expression of enzymes involved in lipid metabolism is also altered and its inhibition could suppress the tumour growth in glioma. Carnitine palmitoyltransferase 1 (*CPT1*), a regulator of long‐chain fatty acid transportation and beta‐oxidation, is elevated at expression level in high‐grade glioma. Glioma cells treated with *CPT1* inhibitor etomoxir exhibited inhibited growth.^22^, ^23^ Acyl‐CoA synthetase homolog 3 (*ACSVL3*), which adds coenzyme A to fatty acids, was also over‐expressed and involved in regulation of self‐renewal maintenance in glioma.^24^, ^25^ Expression of fatty acid synthase (*FASN*) increased with tumour grade, and pharmacological inhibition of *FASN* significantly decreased the proliferation and migration of glioma stem cells.^26^, ^27^ In this study, we profiled the lipid metabolism phenotype between low‐grade glioma and GBM with gene expression data. The results found GBM exhibited enrichment of glycosphingolipid metabolic progress, whereas LGG displayed enrichment of phosphatidylinositol metabolic progress, which offered new implications regarding glioma lipid metabolism status and targeted therapy.

Risk score is a widely used approach to construct a meaningful signature.^28^ A lipid metabolic gene panel could serve as powerful prognostic biomarker and stratify patients for lipid metabolism targeted therapies. In the present study, we profiled the lipid metabolism phenotype in glioma and found distinct lipid metabolism progresses were enriched in LGG and GBM (Figure 1). Fifty‐one differentially expressed genes between LGG and GBM, involved in distinct lipid metabolism progresses (glycosphingolipid metabolic and phosphatidylinositol metabolic progress), were employed to develop a prognostic indictor. Finally, we identified a nine‐gene set that could stratify patients with high‐ or low‐risk of poor prognosis. Moreover, functional analyses revealed that the signature could reproduce the lipid metabolic difference among patients (glycosphingolipid metabolic progress was enriched in high‐risk patients, and low‐risk ones exhibited enrichment of phosphatidylinositol metabolic progress) (Figure 5A,B).

Due to the insufficiency of univariate Cox model for variables selection, we first performed univariate Cox model to acquire genes which were correlated with overall survival and conducted an elastic net regression Cox model to improve the predictive ability of the prognosis.^14^ While none of the obtained nine genes showed high coefficient in Cox model, multiple genes showed a cumulative predictive performance on survival. Most of these genes, such as *CDIPT*, *PIK3C2G*, *ARSJ*, *ARSE*, *GLA* and *GLB2*, had not been studied in cancers. *MTMR7* protein was down‐regulated with increasing tumour grade and stage in colorectal cancer,^29^ while *PIK3CB* 30 and *UGCG* 31, 32 preferentially up‐regulate and promote cancer progression. We further explored the expression and prognostic correlation of these nine genes in TCGA RNA sequencing data. *CDIPT*, *MTMR7*, *PIK3CB* and *PIK3C2B* showed decreased expression in GBM compared with LGG, and their high levels were associated with favourable outcome. In contrast, the other five genes were up‐regulated in GBM, and high expression indicated poor outcome (Figure S8). The biological roles of these nine genes in gliomagenesis need to be further explored.

Since GO and GSEA revealed that high‐risk cases showed an enhanced phenotype of immune response, we also performed the ESTIMATE algorithm to compare inflammatory microenvironment between high‐ and low‐risk groups. Consequently, we found a significant increase in ESTIMATE scores in the high‐risk group (Figure S9), indicating that the lipid metabolism status is associated with inflammatory microenvironment in diffuse gliomas.

## 5 CONCLUSION

Collectively, we profiled the lipid metabolism phenotype in diffuse gliomas and identified a lipid metabolic gene signature that could classify patients with high‐ and low‐risk categories of poor outcome. Our workflow was summarized in Figure S10. However, prospective studies were further needed and the predictive capacity of the gene panel regarding lipid metabolism should be tested for clinical application.

## CONFLICT OF INTEREST

The authors confirm that there are no conflicts of interest.

## AUTHOR CONTRIBUTION

TJ designed the study and wrote the manuscript. FW, RC and ZZ performed the gene analysis. YL, GL and HJ collected the clinical data.

## Data Availability

All data can be downloaded from TCGA database (http://cancergemome.nih.gov/) and CGGA database (http://www.cgga.org.cn).
