# Supplementary material for: Prognostic power of a lipid metabolism gene panel for diffuse gliomas
Source: J Cell Mol Med. 2019 Sep 1;23(11):7741–8. doi: 10.1111/jcmm.14647 (PMC6815778; doi:10.1111/jcmm.14647)
Supplement: Supplementary file 1 [file JCMM-23-7741-s001.docx]

**
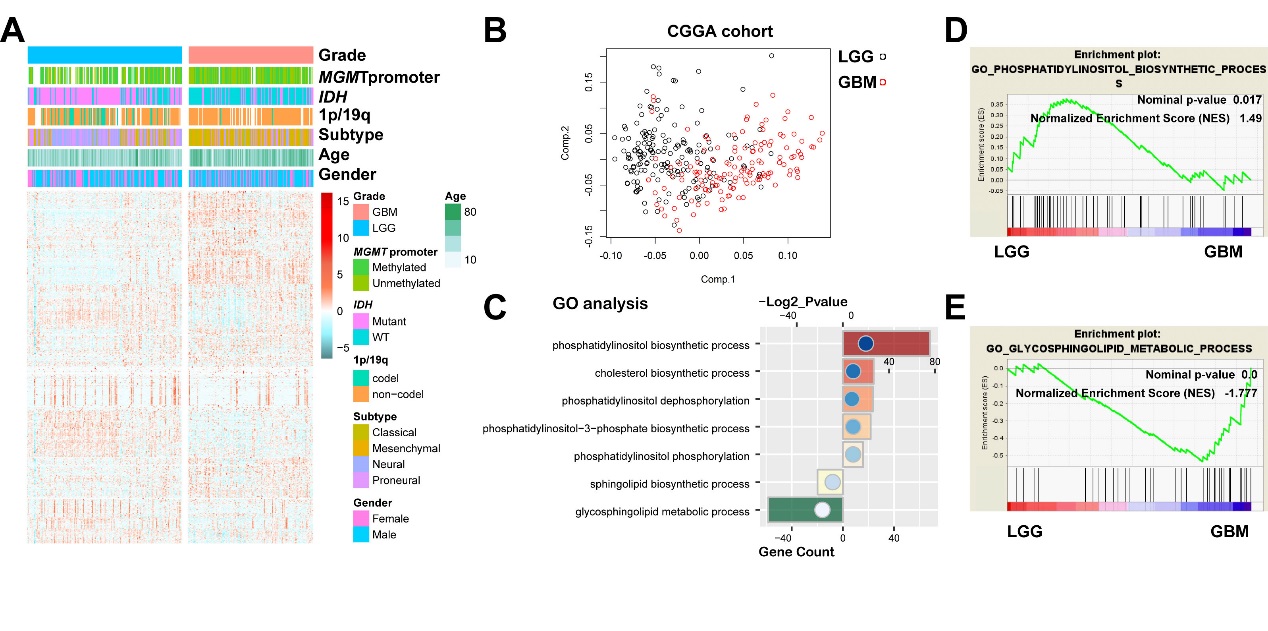
**

**Figure S1.** Distinct lipid metabolism status between LGG and GBM in CGGA cohort. (**A**) Heatmap of lipid metabolism-related genes between LGG and GBM of CGGA cohort. (**B**) Principal components analysis of lipid metabolism-related genes between LGG and GBM. (**C)** GO analysis of differential genes between LGG and GBM. (**D** and **E**) Gene set enrichment analysis of lipid metabolism status between LGG and GBM. NES, normalized enrichment score.


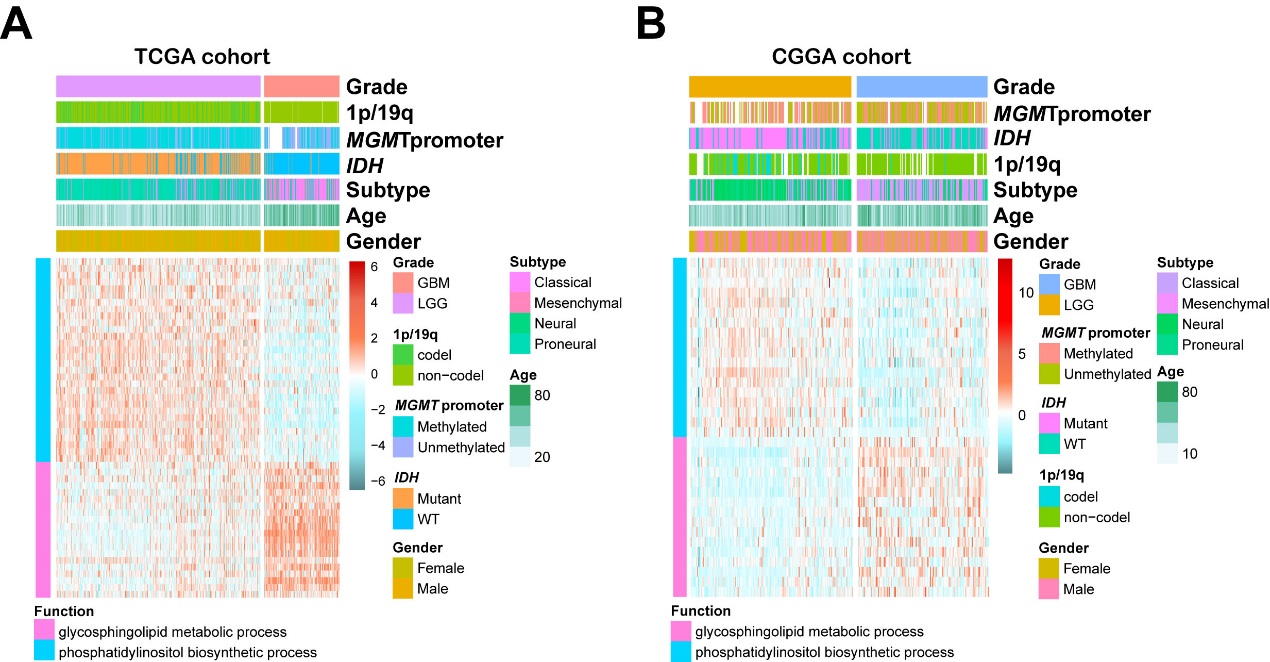


**Figure S2.** Heatmaps show the differential genes between LGG and GBM, involving in glycosphingolipid and phosphatidylinositol metabolic progress. (**A**) TCGA cohort (51 genes). (**B**) CGGA cohort (34 genes).


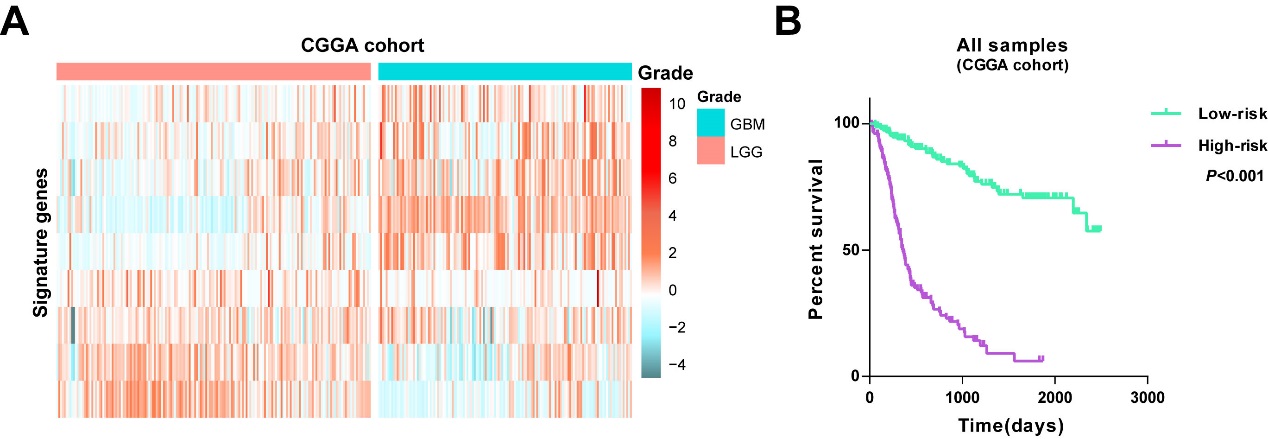


**Figure S3.** Survival analysis of the acquired signature in CGGA cohort. (**A**) Heat map shows the signature genes. (**B**) Survival analysis of OS in high and low-risk groups of patients.


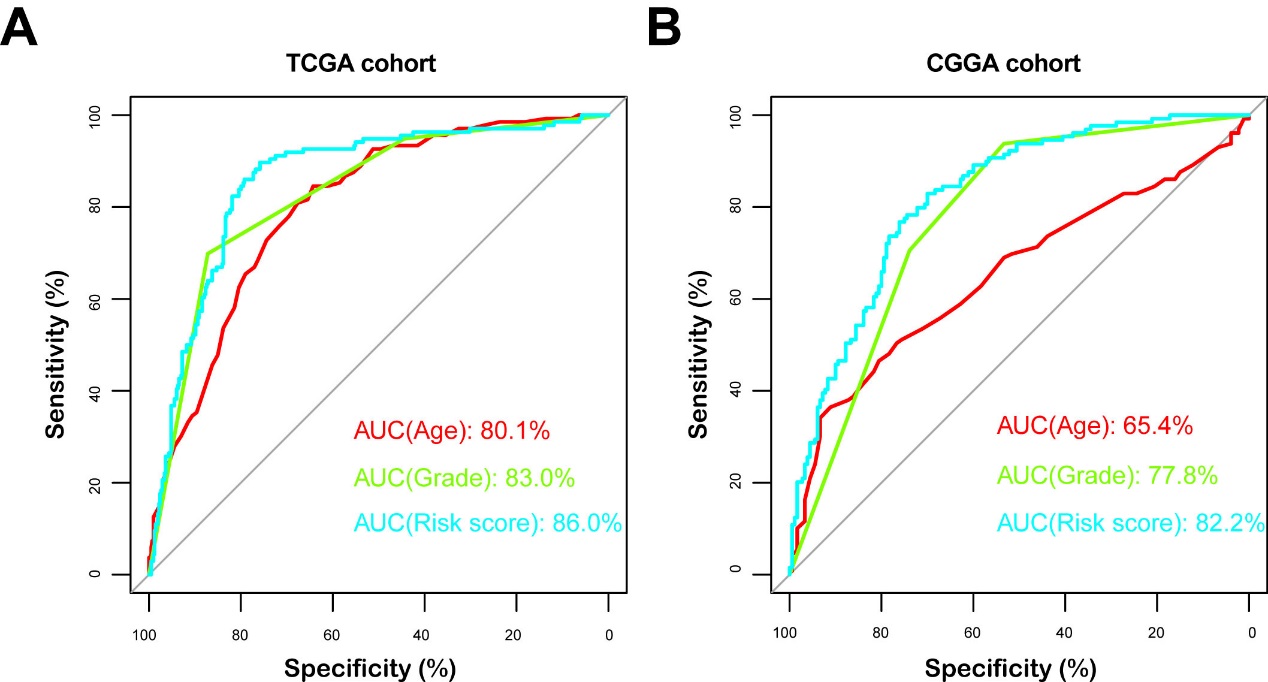


**Figure S4.** ROC analyses in TCGA and CGGA cohorts. (**A** and **B**) ROC curve analysis using age, grade and risk score. AUC, area under the curve.


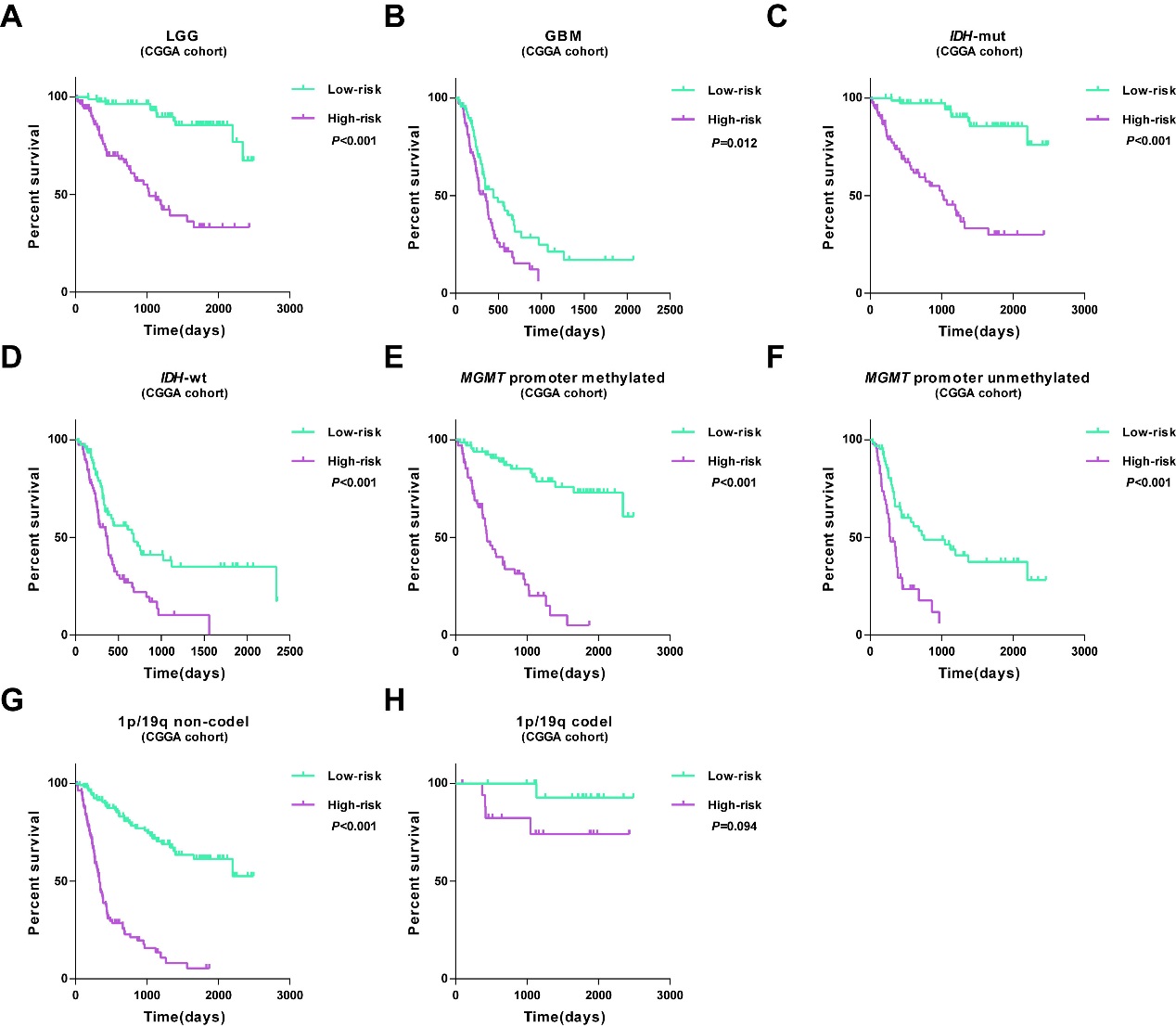


**Figure S5.** Outcome prediction of the signature in stratified patients of CGGA cohort. (**A**-**H**) Survival analysis of the signature in patients stratified by grade, *IDH*, *MGMT* promoter and 1p/19q status.


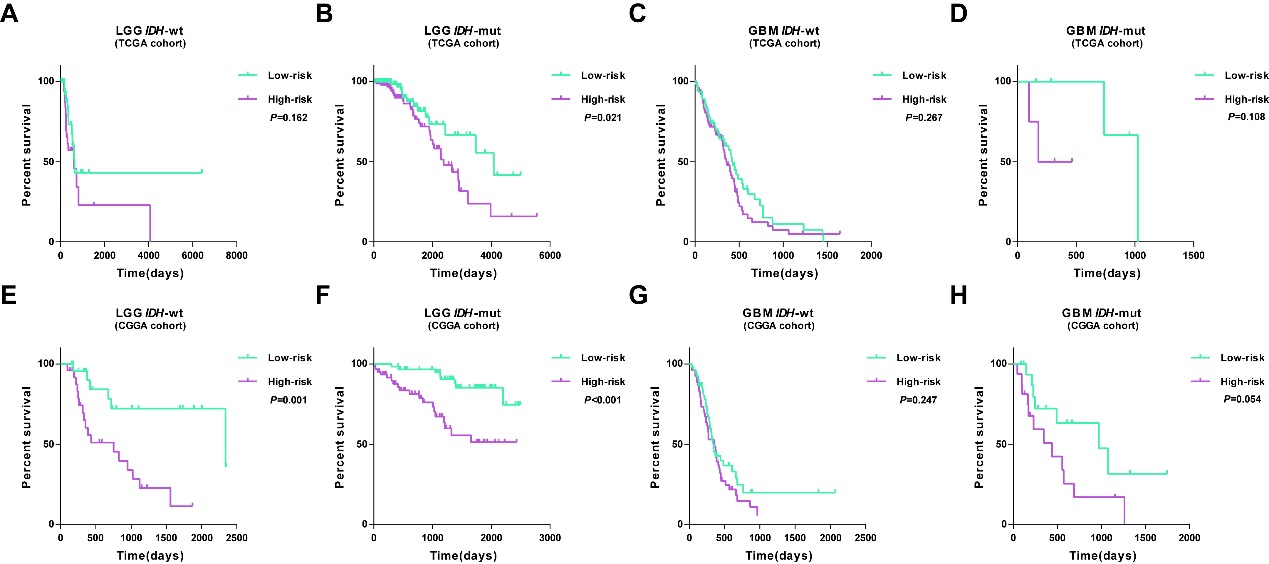


**Figure S6.** Outcome prediction of the signature in patients of different molecular subgroups. (**A**-**D**) Survival analysis in molecular subgroups of TCGA cohort (LGG *IDH*-mut, LGG *IDH*-wt, GBM *IDH*-mut and GBM *IDH*-wt). (**E**-**H**) Survival analysis in molecular subgroups of CGGA cohort (LGG *IDH*-mut, LGG *IDH*-wt, GBM *IDH*-mut and GBM *IDH*-wt).


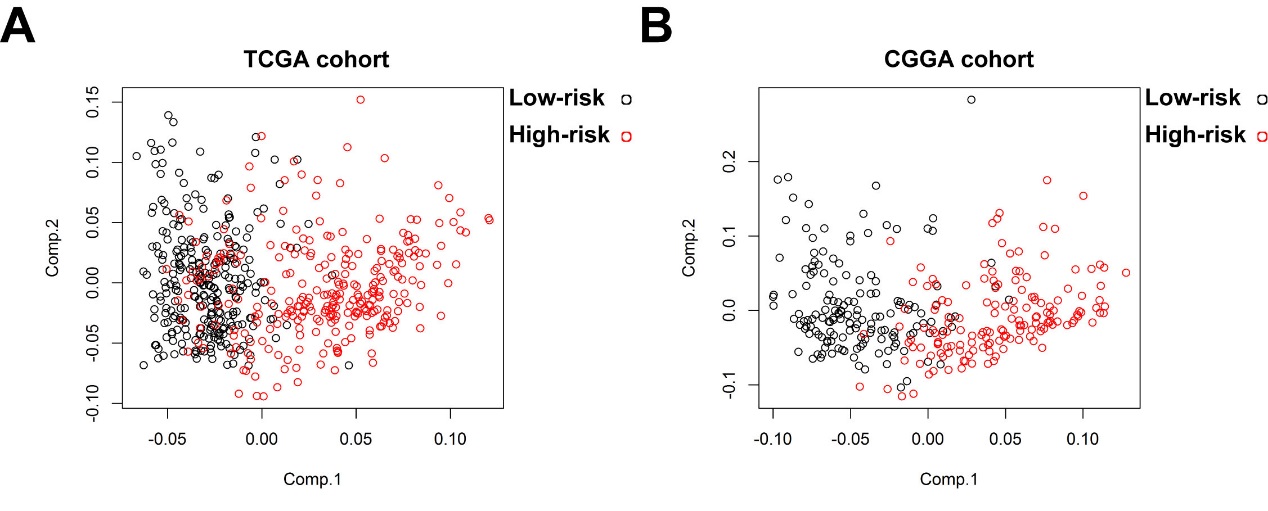


**Figure S7.** Principal components analysis of high and low-risk groups of patients based on whole gene expression data. (**A** and **B**) PCA in TCGA and CGGA cohorts.


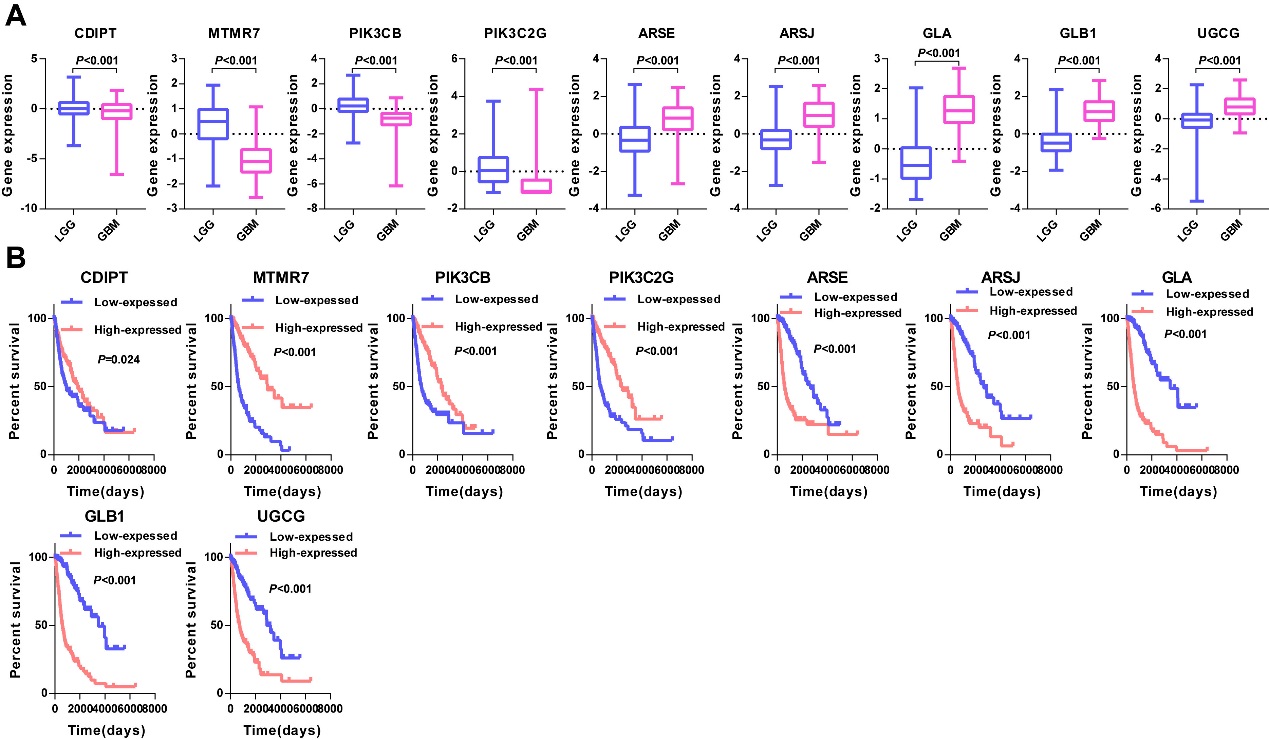


**Figure S8.** Expression and survival analyses of signature genes in RNA sequencing data. (**A**) Expression of signature genes between LGG and GBM. (**B**) Kaplan-Meier analysis of nine genes in diffuse gliomas of TCGA RNA sequencing cohort.


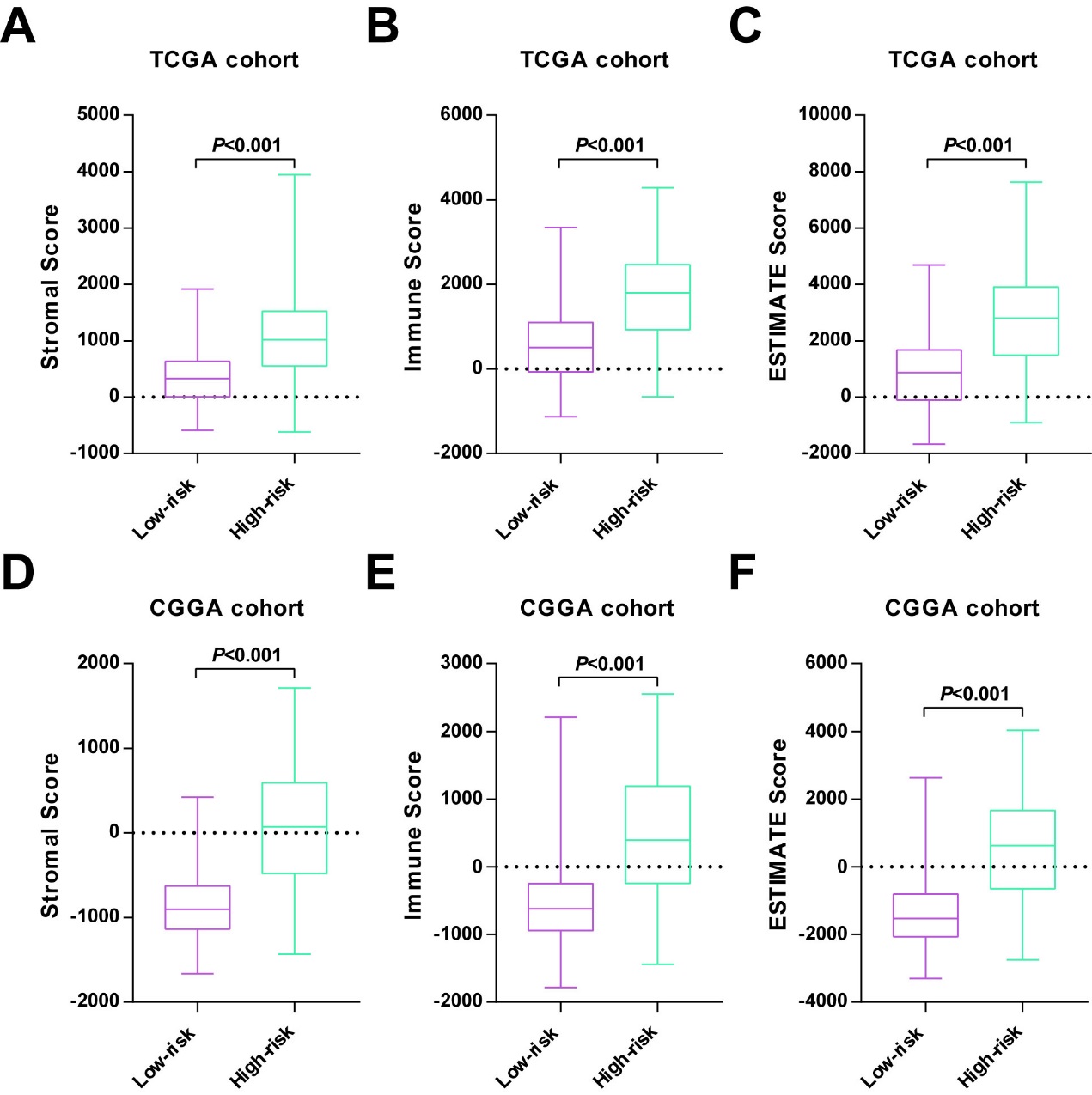


**Figure S9.** Comparison of stromal and immune scores in high and low-risk groups in TCGA and CGGA cohorts. (**A**-**C**) TCGA cohort. (**D**-**F**) CGGA cohort.


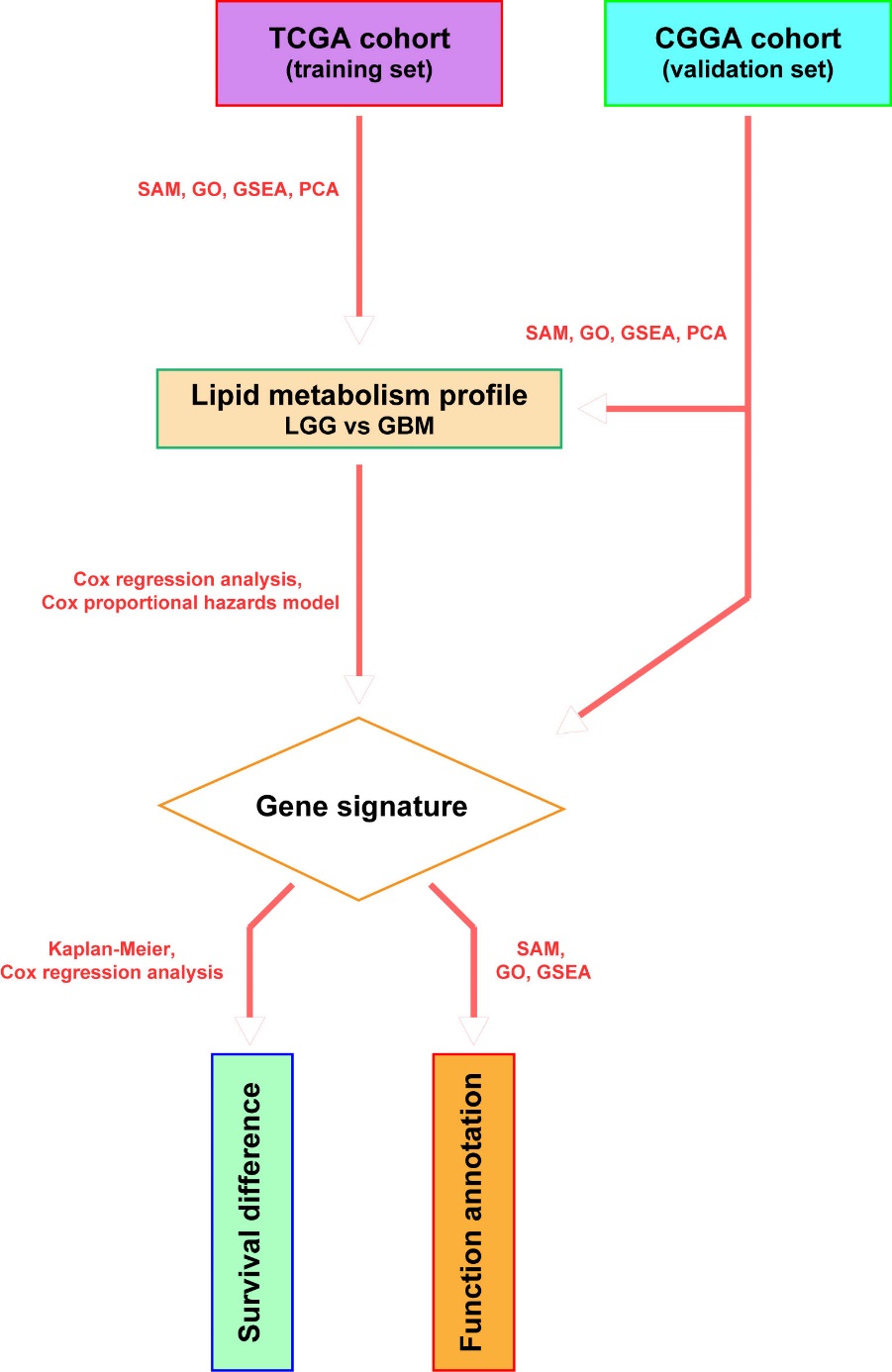


**Figure S10.** Workflow for this study.

We profiled the lipid metabolism status in 859 diffuse glioma samples with gene expression data from TCGA and CGGA database. Additionally, we constructed a risk signature for prognostic prediction with the differential genes involving in glycosphingolipid and phosphatidylinositol metabolic progress.

**Table S1.** Characteristics of patients in low-risk and high-risk groups in TCGA and CGGA cohorts.

| **TCGA cohort** | | | | | **CGGA cohort** | | | | |
| --- | --- | --- | --- | --- | --- | --- | --- | --- | --- |
| **Characteristics** | **n** | **Low-risk** | **High-risk** | ***P*-value** | **Characteristics** | **n** | **Low-risk** | **High-risk** | ***P*-value** |
| **Total Cases** | 550 | 275 | 275 |  | **Total Cases** | 309 | 154 | 155 |  |
| **Age** |  |  |  |  | **Age** |  |  |  |  |
| ≤48 | 287 | 198 | 89 | **<0.001** | ≤43 | 166 | 99 | 67 | **<0.001** |
| >48 | 263 | 77 | 186 |  | >43 | 143 | 55 | 88 |  |
| **Gender** |  |  |  |  | **Gender** |  |  |  |  |
| Male | 319 | 151 | 168 | 0.142 | Male | 194 | 91 | 103 | 0.181 |
| Female | 231 | 124 | 107 |  | Female | 115 | 63 | 52 |  |
| **Subtype** |  |  |  |  | **Subtype** |  |  |  |  |
| Classical | 141 | 2 | 139 | **<0.001** | Classical | 69 | 11 | 58 | **<0.001** |
| Mesenchymal | 31 | 1 | 30 |  | Mesenchymal | 65 | 1 | 64 |  |
| Proneural | 345 | 248 | 97 |  | Proneural | 99 | 73 | 26 |  |
| Neural | 33 | 24 | 9 |  | Neural | 76 | 69 | 7 |  |
| **Grade** |  |  |  |  | **Grade** |  |  |  |  |
| II | 191 | 158 | 33 | **<0.001** | II | 104 | 94 | 10 | **<0.001** |
| III | 211 | 115 | 96 |  | III | 67 | 38 | 29 |  |
| IV | 148 | 2 | 146 |  | IV | 138 | 22 | 116 |  |
| ***IDH*** |  |  |  |  | ***IDH*** |  |  |  |  |
| Mut | 338 | 267 | 71 | **<0.001** | Mut | 155 | 123 | 32 | **<0.001** |
| WT | 212 | 8 | 204 |  | WT | 154 | 31 | 123 |  |
| ***MGMT* promoter** |  |  |  |  | ***MGMT* promoter** |  |  |  |  |
| Methylated | 383 | 251 | 132 | **<0.001** | Methylated | 136 | 72 | 64 | **<0.001** |
| Unmethylated | 135 | 24 | 111 |  | Unmethylated | 111 | 34 | 77 |  |
| NA | 32 | 0 | 32 |  | NA | 62 | 48 | 14 |  |
| **1p/19q** |  |  |  |  | **1p/19q** |  |  |  |  |
| Codeleted | 137 | 110 | 27 | **<0.001** | Codeleted | 157 | 32 | 125 | **<0.001** |
| Non-codeleted | 407 | 165 | 242 |  | Non-codeleted | 101 | 97 | 4 |  |
| NA | 6 | 0 | 6 |  | NA | 51 | 25 | 26 |  |

*IDH*, isocitrate dehydrogenase; *MGMT*, methylguanine methyltransferase.
